# Migration and sociodemographic factors associated with sense of place attitudes among migrants of African background in Northern and Western Netherlands

**DOI:** 10.1002/jcop.22118

**Published:** 2018-08-24

**Authors:** Victor Counted, Ahmed Moustafa, Andre Renzaho

**Affiliations:** ^1^ Western Sydney University Australia; ^2^ University of Groningen Netherlands

## Abstract

This article investigates migration and sociodemographic factors associated with attitudes toward specific geographic settings (place attachment [PA], place identity, place dependence [PD]) in a cross‐sectional data (N = 175) of sub‐Saharan African residents in the Northern Netherlands and the Western Netherlands. Overall, it was found that scores of PA and PD were stronger in long‐term African residents (more than 5 years) than among short‐term residents (less than 5 years). The region of residence was positively related to PD, with participants in western Netherlands having stronger PD compared to those in the northern region. Language proficiency was inversely associated with PD among participants with a little knowledge of Dutch compared to those who could speak Dutch fluently. Older African residents (aged 46–55 years) were more likely to develop PA and PD than their younger counterparts (aged 18–25 years). PA scores were significantly lower for participants with a tertiary education background compared to those with a high school education or less educational attainment. PD scores were lower in females than males, and participants from Western Africa had stronger levels of PD compared to those from Central Africa. Implications of these findings are discussed.

## 1 THEORETICAL AND EMPIRICAL BACKGROUND

Netherlands is divided into four regions: southern, northern, eastern, and western. Although there are similarities and distinctions between these regions, most cities and provinces in the western and northern regions of the Netherlands are considered multicultural cities with a significant number of international migrants, whereas cities in the southern and eastern regions are quite unlike the rest of the country in that they have a smaller migrant population (Nabialek, Hamers, & Evers, 2016). The western and northern regions of the Netherlands have well‐known cities such as Amsterdam, Den Haag, Utrecht, Rotterdam, Leiden, Zwolle, and Groningen, renowned for institutions such as the University of Amsterdam, University of Groningen, and Universiteit Utrecht. These institutions are largely responsible for attracting thousands of international migrants, working professionals, and students to the Western Netherlands and the Northern Netherlands, making these regions in the Netherlands a melting pot of culture and the largest metropolitan areas attracting people of diverse backgrounds (Nabialek et al., 2016).

A recent report by Statistics Netherlands (2016) suggested a 0.5% growth in the estimated number of inhabitants in the Netherlands (about 16.9 million). Statistics Netherlands attributed this growth to the 202,647 migrants registered at a Dutch municipality (mostly in the western and northern regions) within the past two decades, with an expected number of about 20,000 newly arrived migrants each year. Of the 16.9 million inhabitants in the Netherlands, sub‐Saharan Africans make up about 1.30% of this population, hence a significant population from which to generate knowledge to inform policy and practice. Although the statistics on the migration of people to the Netherlands parallel with the growing trend on migration across other parts of western Europe (e.g., De Haas, 2008; Gemmeke, 2013; Van Hear, 1998), little is known about how migrants, particularly Africans, are settling in their new abodes in terms of developing a sense of place (SOP), in relation to feelings of attachment to, identity of, and dependence on a new place, despite the increasingly antimigrant climate across Europe (Gemmeke, 2013). SOP theory has great implication for migration, and the analytical potential of this concept is yet to be fully realized as far as the scholarly study of migration is concerned (Mendoza & Moren‐Alegret, 2012).

The migratory patterns of people who migrated from Africa to the Netherlands require a closer look, such that one considers how people of African descent develop their SOP in a Dutch society and the determinants related to their place experiences in metropolitan areas such as the northern and western parts of the Netherlands. The experience of SOP can be complex, especially among migrant groups like the African diasporas, because it can be strongly influenced by culture (e.g., Dutch individualism vs. African migrant collectivism), pre‐ and postmigration factors (e.g., migration status, refugees vs. skilled migrants), and settlement experiences (e.g., discrimination and past and present social and emotional experiences). Besides, studies looking at sense of belonging rarely examine migration‐related and sociodemographic factors, a gap the current study seeks to address. Thus, we aimed to study people's attitudes toward the Dutch society by exploring which migration and sociodemographic factors may serve as predictors of SOP experiences among African residents in the Western Netherlands and the Northern Netherlands.

### 1.1 Sense of place: theory and research

According to Jorgensen and Stedman (2001), SOP refers to not only attachment and dependence but also forming an identity with the same space (or spatial setting). Accordingly, SOP is a general term that measures three different but related concepts: place identity (PI), place dependence (PD), and place attachment (PA; Jorgensen & Stedman, 2001). The multidimensionality of place theory positions how individuals can experience place at three domains (Counted, 2016). These place experiences are conceptualized, based on the sense of place attitude theory, as the responses to an object or stimulus in a geographic setting that has the potential to arouse attachment to the place, identity of that place, and behavioral commitment in that place (Counted, 2016; Jorgensen & Stedman, 2001).

PI, based on Counted's (2016) multidimensional approach to place, represents the personal dimension of a place which has a cognitive function, and reflecting individual or group's beliefs, perceptions, character, or identity that the self has developed in a particular place (e.g., accent or lifestyle of a place). PA refers to attachment, often in the form of an attraction, to the physical and natural elements of a place, thus playing an affective role in drawing the individual or group to a particular geographic setting (such as Christians to Jerusalem or Muslims to Mecca). Counted (2016) conceptualizes this as the place dimension or attachment to the environmental ensemble in his multidimensional approach to place theory. PD represents the psychological process dimension in place theory, which suggests “an ongoing relationship with a place that supports our life goals and activities” (Counted, 2016, p.13). Each of the three aspects emphasizes domains of place that are involved in people–place relationships and experiences.

Proshansky, Fabian, and Kamino (1983) noted that PI is analogous to gender or role identity in that one can identify with what they are living (e.g., one may say “I am a New Yorker,” which shows a stronger PI than “I live in New York”). On the other hand, PA refers to affective or emotional processes toward a spatial setting (Williams, Patterson, Roggenbuck, & Watson, 1992). PA is analogous to concepts, such as child–parent attachment. PD is a psychological process toward a place, yet unlike PA, it can be negative in the event of forced displacement when the individual depends on the resources of a place for survival. PD is possibly similar to behavioral dependence in that one cannot break certain behaviors. In a subsequent study, Jorgensen and Stedman (2006) investigated the factors underlying SOP among lakeside property owners in North Wisconsin. They found that time spent at the property and perception of environment nearby, including the lake itself as well as age of the property owner, plays a role in forming a SOP.

According to several studies, psychological dimensions do play a key role in the formation and development of SOP (Ujang & Zakariya, 2015). Along these lines, it is suggested that having memories associated with a spatial setting also is related to the strength of SOP and its subcomponents, PA, PI, and PD (Gustafson, 2001). Having or forming a SOP is quite important because it helps with the management of the environment and spatial setting. Chigbu (2013) argued that a lack of SOP among rural residents in Uturu, Nigeria, is detrimental to development. Along these lines, Hay (1998) reported that SOP is related to being raised in a place, and this is even stronger for indigenous people, such as the Maori in New Zealand.

### 1.2 Migration factors, sociodemographic status, and sense of place

Because migration‐related factors seem to play a vital role in influencing SOP, the understanding of SOP in people's lives provides a new way of thinking about migration. Kohlbacher, Reeger, and Schnell (2015) pointed this out in their study with migrants in Vienna and Austrian natives of Vienna, reasoning that migration factors such as place of origin and residence are more important in explaining people–place experiences than sociodemographic factors. Their study shows that Vienna natives had a stronger SOP compared with migrants, who were significantly less drawn to Austria. This dichotomy was linked to deprivation and mobility among migrants (who lived in deprived settings) and social ties among local natives (who lived in neighborhoods that were better off).

Geographic mobility was also implicated in the experience of SOP among migrants and returnees in Chaohu, China (Du, 2015). Bailey, Kearns, and Livingston (2012) showed that local natives and migrants may have different patterns of experiences in a place. For instance, the SOP is significantly lower in migrant groups because areas where most migrants live are likely to have a weaker social cohesion, even though the drivers of attachment may still remain the same between the natives and migrants.

The place of residence in a multicultural country also emerged as an important factor among urban and rural residents in Western Australia (Anton & Lawrence, 2014), pointing to the residential neighborhood as an important migration variable in studying SOP. A similar study was conducted in a Dutch context, in which researchers examined people–place relationships in deprived neighborhoods among Dutch citizens (Lager, van Hoven, & Meijering, 2012; van der Graaf, 2009). Lager et al. (2012) suggest that the place of residence (e.g., in co‐housing communities) serves as a central setting of experience from which migrants explored their broader environment and formed new attachments in the community.

Immigration and citizenship status have been linked to SOP and feeling of belonging. For example, Simonsen (2017) found that having a residence visa status and citizenship were associated with increased national belonging (PI or PA) in developed countries where citizenship is a mark of national membership, compared with developing countries where citizenship has less important. Brettell (2006) has also positioned immigration status as a significant determinant of SOP and belonging in that it helps to shape attitudes toward PI and citizenship, arguing that migrants have a bifocal outlook on SOP.

Other studies have suggested that the individual's length of stay in a particular geographic setting can be a strong predictor of SOP (Anton & Lawrence, 2014; Vaske & Kobrin, 2001). Individuals who have stayed longer in a place are likely to have stronger SOP compared with those who are fairly new to the place. The same argument was made on the relationship between SOP and the language proficiency of a place, which can be mediated by the length of stay. Environmental psychologists Anton and Lawrence (2014) explain: “The longer a person stays in a place the greater the likelihood of the place being incorporated into the identity structure, especially if that place also provides the individual with feelings of distinctiveness, continuity, self‐esteem and self‐efficacy” (p.452). This is supported by previous studies conducted by Moore and Graefe (1994) and Vaske and Kobrin (2001), which theorize that PD tends to precede PI. This is because when a particular place meets the individual's needs, they are likely to stay longer there and in the process of depending on the place, they could form an attachment to and identity of that place. Therefore, individuals who have stayed longer in a place are likely to have learned the language of their place of residence, which can be conceptualized as a form of PI because language essentially represents the identity of a place.

Along with migration factors, sociodemographic variables such as gender differences have been postulated to account for the variance in SOP. For example, Rose, Carrasco, and Charboneau (1998) found that females have weak ties to place compared with their male counterparts. This was reported in other similar studies (e.g., Campbell & Lee, 1990; Manzo, 2003), which concluded that women tend to experience place differently from men. A study by Du (2015) suggests that educational attainment is related to SOP in migrants and returnees in China. Du also found that educated migrants tend to have a stronger SOP in a host city and are likely to be more welcomed and accepted by the new sociospatial environment than noneducated migrants. Lewicka (2010) argued that age and level of education are important factors necessary for studying place experiences. It is also possible that older people are more likely to have stronger SOP than young people due to the years living in a place.

The above‐mentioned studies point to the prevailing effect of migration and sociodemographic factors on SOP while revealing both positive (Forrest & Kearns, 2000; Greif, 2009; Gorney & Rounczyk‐Ruiz, 2014) and negative outcomes of SOP in terms of intergroup conflicts (Devine‐Wright, 2009; Fried, 2000) and the lack of desire to explore other places (Twigger‐Ross & Uzzell, 1996), among others. Interestingly, none of the studies on SOP in Europe has specifically covered the experience of the sub‐Saharan African migrant group living in foreign countries, except for those done in England (Waite & Cook, 2011) and the United States (Rawlings, 2010).

A lack of empirical data on African migrants is observed in research on SOP in the Netherlands and Europe in general. It is worth mentioning that the work of Buitelaar and Stock (2016) on attachment to “home'” among Moroccan Dutch citizens may also reflect the SOP experiences of North African migrants in a Dutch society. Studying the SOP of migrant groups in host countries has important implications for migration studies in terms of understanding how such groups feel at home in a foreign country and the sociodemographic and migration experiences that facilitate their civic integration. These experiences, it is believed, are the window to exploring aspects of place experiences that are developed as determinants of migration and sociodemographic factors. Therefore, this study investigates how SOP (PA, PI, and PD) is related to migration and sociodemographic factors; thus the following hypotheses are examined:

H1: Migration variables such as region of residence, length of stay, language proficiency, and immigration status will be statistically related to SOP attitudes (PA, PI, and PD).
H2: Sociodemographic factors such as age, gender, relationship status, level of education, and religious background will be statistically associated with SOP attitudes (PA, PI, and PD).

## 2 METHOD

### 2.1 Procedures

The faculty board of the University of Groningen approved this research. We obtained informed consent from participants with a sub‐Saharan African background to conduct a cross‐sectional survey between November 2015 and April 2016. The participants were recruited from Amsterdam, Groningen, Utrecht, Rotterdam, Almere, Den Hague, and Assen, which are cities in the Western Netherlands and the Northern Netherlands with the largest population of international migrants. Cross‐sectional data were collected from 175 African residents who had settled in northern and western Netherlands.

The study used a snowball sampling approach–a method used for recruiting the participants from existing community structures. Renzaho and colleagues (2006, 2008, 2009, 2012, 2014) conducted a series of studies in which the viability of using the snowball sampling technique among African migrants was examined. The authors noted that the snowball technique for research on African migrants is preferable because they are a hard‐to‐reach population and are often excluded in mainstream research because of difficulties accessing them. They concluded that African migrants may be reached only through recommendation from exiting community leaders or structures. They added the advantage of using the snowball sampling technique includes the ability to locate hidden and minority populations and hence the opportunity to fill in the gap in knowledge that cannot be generated by the mainstream research.

Participants had regular meetings as religious groups, student groups, and cultural groups. The first author (VC) toured around the western and northern regions of the Netherlands to participate in different cultural, student, and religious events organized by group leaders, most of whom have been in contact with VC and have been informed about the study. Leaders of these groups informed their respective communities about the study, and a date for the data collection was arranged. Upon arrival at the group events, VC was asked to explain the study to members of the groups. They were told that the study was exploring to what extent their SOP in the Netherlands was influenced by their migration background and sociodemographic status. A total of 353 participants responded to the initial invitation (collecting copies of the surveys) to participate in the study at different meetings and events, but only 175 participants returned their completed surveys at the same time of initial meeting, the following week, or via mail.

### 2.2 Participants

Our participants had been residents of cities in the northern and western parts of the Netherlands for more than 6 months (33.3% and 66.7% respectively): 58.9% far more than 5 years and 41.1% less than 5 years. The participants are originally from Eastern Africa (11.7%), Central Africa (6.9%), Western Africa (73.1%), and Southern Africa (8.3%). They are citizens of the following countries: Angola, Botswana, Burundi, Cameroon, Congo, Eritrea, Gambia, Ghana, Kenya, Lesotho, Nigeria, Sierra Leone, South Africa, Sudan, Tanzania, Uganda, Zambia, and Zimbabwe. Roughly half (52%) were female, and 81.9% of the total sample spoke Dutch or had a little knowledge of the language. Of the participants, 72.4% were between 18 to 45 years of age, with the remaining older than age 45 years. In the total sample, 56.3% had completed tertiary education (compared with 43.7% with high school or less education); 50.6% were married, 43.1% were single, and 6.3% were divorced, widowed, or separated. Almost all the participants (94.2%) were Christian, 3.5% Muslim, 1.2% African traditional religious followers, and 1.2% identified their religion as other.

### 2.3 Measures and variables

#### 2.3.1 Outcome variables

##### SOP experiences

We used a 12‐item measure, adapted from the Sense of Place Scale by Jorgensen and Stedman (2001), to assess the SOP of the participants in terms of their PA, PI, and PD. The measurement was guided by the three‐factor model for measuring SOP, which is seen as a response to an object or stimulus in a place that has the potential to arouse attachment, identity, and behavioral commitment (dependence; Jorgensen & Stedman, 2001).

PA (α = 80) refers to the physical attraction to a place or attachment to “human‐made and non‐human‐made material elements and environmental qualities of a particular place” (Jorgensen & Stedman, 2001, p.13). PI (α = .61) refers to an individual or group's “cognitions, beliefs, perceptions or thoughts that the self has invested in a particular spatial setting” (Jorgensen & Stedman, 2001, p.238), showing how a place can function as a repository for emotions and relationships that give meaning to life in a particular place. PD (α = .67), on the other hand, is “an ongoing relationship with a place that supports our life goals and activities” (Counted, 2016, p.13).

Each of the three aspects–PA, PI, and PD–describing the dimensions of SOP were used in this study, along with the overall 12 items SoP Scale, which were combined to measure a composite of general SOP (α = .83). Participants rated the items on a 5‐point scale ranging from 1 (*disagree strongly*) to 5 (*agree strongly*), with higher (mean) scores suggesting higher levels of the construct in each dimension. Two negatively worded PD items were reverse coded before data analysis.

#### 2.3.2 Predictor variables

##### Migration factors

The variables are as follows: place or region of residence (0 = Northern Netherlands, 1 = Western Netherlands); length of stay (0 = less than 5 years, 1 = more than 5 years); and Dutch language proficiency (0 = yes speaks Dutch, 1 = do not speak Dutch, 2 = a little knowledge of Dutch). Immigration status was measured with the question, “Do you currently hold a (valid) residence permit for the Netherlands?” (0 = yes, have a valid residence permit, 1 = no valid residence permit, 2 = do not want to disclose).

#### 2.3.3 Sociodemographic factors

The variables are as follows: age (0 = 18–25, 1 = 26–35, 2 = 36–45, 3 = 46–55, 4 = ≥ 56 years), gender (0 = female, 1 = male); relationship status (0 = married, 1 = single [or not in a long‐term relationship], 2 = separated/widowed/divorced); level of education (0 = high school or less, 1 = tertiary education); religious background (0 = Christian, 1 = Muslim, 2 = African Traditional Religion, 3 = other); and region/place of origin in Africa (0 = Central, 1 = Eastern, 2 = Western, 3 = Southern).

##### Statistical Analysis

Study variables in Table 1 show satisfactory Cronbach's alphas. Data were analyzed using STATA (version 13). In Table 1, we provided a descriptive summary of the SOP, migration, and sociodemographic data with the percentiles and variable means. We conducted univariate (UB, unadjusted model) and multivariate (AB, adjusted model) analyses using the hierarchical regression analysis to examine the main effects of sociodemographic and migration factors on SOP experiences (PA, PI, and PD; see Table 2). All control variables from the sociodemographic and migration variables with a *p* < .10 were entered in the adjusted model (AB) examining the associations between sociodemographic and migration factors and SOP. Significance was set at *p* < 0.05.

**Table 1:** Descriptive statistics for study sample (N 175)

SOP variables	α	M	SD	Min	Max
General SOP	.83	3.0817	0.51681	1.83	5
PA	.80	3.2243	0.80252	1	5
PI	.61	3.1668	0.72454	1	5
PD	.67	2.9634	0.74871	1	5
**Migration and sociodemographic variables**	N	%			
***Immigration status***					
Yes, have a valid residence permit	154	89.5%			
No valid residence permit	13	7.6%			
Do not want to disclose 5 (2.9%)	5	2.9%			
***Regions of residence in the Netherlands***					
Northern Netherlands	54	33.3%			
Western Netherlands	108	66.7%			
***Dutch language proficiency***					
Speak Dutch	84	48.8%			
Do not speak Dutch	31	18%			
A little knowledge of Dutch	57	33.1%			
***Age***					
18‐25	34	19.5%			
26‐35	39	22.4%			
36‐45	53	30.5%			
46‐55	26	14.9%			
> = 56 years	22	12.6%			
***Gender***					
Males	83	48%			
Females	80	52%			
***Region of birth in Africa***					
Central Africa	10	6.9%			
Eastern Africa	17	11.7%			
Western Africa	106	73.1%			
Southern Africa	12	8.3%			
***Relationship status***					
Married	88	50.6%			
Single	75	43.1%			
Separated/divorced/widowed	11	6.3%			
***Level of education***					
High school or less	76	43.7%			
Tertiary education	98	56.3%			
***Length of stay in the Netherlands***					
Less than 5 years 72 (41.1%)	72	41.1%			
More than 5 years 103 (58.9%)	103	58.9%			
***Religious background***					
Christianity	163	94.2%			
Islam	6	3.5%			
African Traditional Religion	2	1.2%			
Other	2	1.2%			
***Length of stay in the Netherlands***					
Less than 5 years	72	41.1%			
More than 5 years	103	58.9%			
***Religious background***					
Christianity	163	94.2%			
Islam	6	3.5%			
African Traditional Religion	2	1.2%			
Other	2	1.2%			

*Note*. SOP = sense of place; M = mean; SD = standard deviation; PS = place attachment; PI = place identity, PD = place dependence.

**Table 2:** Regression models for SOP variables [95% confidence interval] by sociodemographic and migration factors

	PI		PD		PA		SOP	
Migration factors	UB [95% CI]	AB [95% CI]	UB [95% CI]	AB [95% CI]	UB [95% CI]	AB [95% CI]	UB [95% CI]	AB [95% CI]
***Dutch region of residence***								
*Northern Netherlands*	***Ref***	***Ref***	***Ref***	***Ref***	***Ref***	***Ref***	***Ref***	***Ref***
Western Netherlands	.121 [−.10, .34]	0.248 [−.95, 1.44]	1.256 [.26, 2.25]^a^	1.281 [−.02, 2.58]^a^	0.923 [−.13, 1.99]	0.086 [−1.46, 1.63]	2.268 [−.10, 4.64]	1.615 [−1.73, 4.96]
***Length of stay***								
Less than 5 years	***Ref***	***Ref***	***Ref***	***Ref***	***Ref***	***Ref***	***Ref***	***Ref***
More than 5 years	−0.083 [−.34, .17]	−.110 [−.52, .30]	1.646 [.77, 2.52]^a^	0.105 [−1.10, 1.31]	1.425 [.47, 2.78]^a^	0.304 [−.07, .67]	2.996 [.87, 5.12]^a^	0.280 [−.65, 1.21]
***Dutch language proficiency***								
*Speak Dutch*	***Ref***	***Ref***	***Ref***	***Ref***	***Ref***	***Ref***	***Ref***	***Ref***
Do Not Speak Dutch	−0.109 [−.41, .19]	−0.302 [−1.67, 1.06]	−1.195 [−2.42, .03]	−1.109 [−2.59, .38]	−0.906 [−2.25, .44]	−0.494 [−2.27, 1.28]	−2.371 [−5.34, .60]	−1.904 [−5.73, 1.92]
A Little Knowledge of Dutch	0.006 [−.24, .25]	−0.183 [−1.22, .86]	−0.992 [−1.99, −.01]^a^	−1.047 [−2.18, .08]	−0.172 [−1.27, .92]	−0.109 [−1.46, 1.24]	−1.221 [−3.64, 1.20]	−1.339 [−4.25, 1.57]
***Immigration status***								
*Valid residence permit*	***Ref***	***Ref***	***Ref***	***Ref***	***Ref***	***Ref***	***Ref***	***Ref***
No valid residence permit	−0.008 [−1.33, 1.31]	0.050 [−1.62, 1.72]	−0.241 [−1.91, 1.42]	−0.274 [−2.09, 1.54]	−0.608 [−2.41, 1.19]	−0.895 [−3.07, 1.27]	−0.857 [−4.77, 3.05]	−1.1203 [−5.80, 3.56]
Do not want to disclose	1.874 [−.21, 3.96]	1.855 [−.82, 4.53]	1.236 [−1.39, 3.86]	1.1304 [−1.78, 4.04]	2.574 [−.26, 5.41]	2.097 [−1.37, 5.57]	5.684 [−.47, 11.83]	5.083 [−2.41, 12.57]
**Sociodemographic factors**								
***Age***								
*18–25*	***Ref***	***Ref***	***Ref***	***Ref***	***Ref***	***Ref***	***Ref***	***Ref***
26–35	−0.514 [−1.63, .60]	−0.850 [−2.36, .66]	0.043 [−1.32, 1.40]	−0.162 [−1.81, 1.48]	−0.073 [−1.54, 1.39]	0.284 [−1.68, 2.25]	−0.544 [−3.80, 2.71]	−0.727 [−4.96, 3.51]
36–45	−0.305 [−1.35, .74]	−0.540 [−2.11, 1.03]	−0.026 [−1.30, 1.24]	−0.794 [−2.49, .91]	0.247 [−1.12, 1.62]	0.469 [−1.56, 2.50]	−0.082 [−3.13, 2.97]	−0.865 [−5.25, 3.52]
46–55	0.157 [−1.08, 1.39]	−0.207 [−2.12, 1.70]	1.568 [.06, 3.08]^a^	0.318 [−1.76, 2.40]	1.990 [.37, 3.61]^a^	2.426 [−.05, 4.90]^a^	3.715 [.10, 7.33]^a^	2.537 [−2.81, 7.89]
> = 56 years	−0.238 [−1.54, 1.07]	−0.730 [−3.06, 1.60]	1.759 [.17, 3.34]^a^	0.186 [−2.35, 2.72]	1.557 [−.15, 3.26]	1.346 [−1.68, 4.37]	3.078 [−.72, 6.87]	0.802 [−5.73, 7.33]
***Gender***								
*Male*	***Ref***	***Ref***	***Ref***	***Ref***	***Ref***	***Ref***	***Ref***	***Ref***
Female	−0.133 [−.85, .59]	−0.147 [−1.14, .85]	−1.182 [−2.07, −.30]^a^	−1.182 [−2.35, 2.72]^a^	−0.215 [−1.19, .76]	−0.179 [−1.47, 1.11]	−1.532 [−3.66, .61]	−1.578 [−4.37, 1.21]
***Education***								
*High school or less*	***Ref***	***Ref***	***Ref***	***Ref***	***Ref***	***Ref***	***Ref***	***Ref***
Tertiary education	−0.550 [−1.27, .17]	−0.147 [−1.33, 1.04]	−0.648 [−1.55, .25]	0.602 [−.69, 1.89]	−1.372 [−2.32, −.42]^a^	−0.485 [−2.03, 1.06]	−2.570 [−4.70, −.45]^a^	−0.029 [−3.36, 3.30]
***Relationship***								
*Married*	***Ref***	***Ref***	***Ref***	***Ref***	***Ref***	***Ref***	***Ref***	***Ref***
Single	−0.141 [−.89, .61]	0.149 [−1.06, 1.36]	−0.467 [−1.40, .46]	0.521 [−.80, 1.84]	−0.444 [−1.44, .55]	0.459 [−1.11, 2.03]	−1.053 [−3.27, 1.16]	1.131 [−2.26, 4.52]
Separated/widowed/divorced	0.317 [−1.20, 1.84]	1.059 [−1.18, 3.30]	−0.077 [−1.97, 1.82]	0.123 [−2.31, 2.55]	1.452 [−.57, 3.48]	1.684 [−1.22 −4.58]	1.692 [−2.82, 6.20]	2.866 [−3.39, 9.12]
***Region/place of origin***								
*Central Africa*	**Ref**	**Ref**	**Ref**	**Ref**	**Ref**	**Ref**	**Ref**	**Ref**
Eastern Africa	0.566 [−1.32, 2.46]	0.880 [−1.43, 3.19]	−0.832 [−3.03, 1.36]	−0.531 [−3.04, 1.98]	−0.082 [−2.62, 2.45]	0.352 [−2.64, 3.35]	−0.349 [−5.88, 5.18]	0.702 [−5.76, 7.17]
Western Africa	1.013 [−.56, 2.58]	1.201 [−.80, 3.20]	2.064 [.241, 3.89]^a^	2.702 [.53, 4.88]^a^	1.741 [−.36, 3.85]	1.718 [−.88, 4.31]	4.819 [.23, 9.41]^a^	5.620 [.02, 11.22]^a^
Southern Africa	0.781 [−1.25, 2.81]	1.494 [−1.14, 4.13]	−0.971 [−3.33−1.39]	0.821 [−2.04, 3.69]	−0.587 [−2.78, 2.66]	0.976 [−2.44, 4.40]	−0.249 [−6.19, 5.70]	3.291 [−4.09, 10.67]
***Religious background***								
*Christian*	***Ref***	***Ref***	***Ref***	***Ref***	***Ref***	***Ref***	***Ref***	***Ref***
Muslim	−0.815 [−2.81, 1.18]	2.368 [−.86, 5.59]	−0.219 [−2.69, 2.25]	4.083 [.58, 7.59]^a^	−0.297 [−2.94, 2.35]	1.589 [−2.59, 5.77]	−1.331 [−7.21, 4.55]	8.041 [−.99, 17.07]
African Traditional Religion	−0.648 [−4.07, 2.77]	−0.746 [−4.49, 3.00]	−2.885 [−7.11, 1.34]	−2.102 [−6.17, 1.97]	−3.463 [−7.99, 1.06]	−3.163 [−8.02, 1.69]	−6.998 [−17.06, 3.06]	−6.011 [−16.49, 4.47]
Others	−1.148 [−4.57, 2.27]	−0.949 [4.80, 2.90]	−1.385 [−5.61, 2.84]	0.974 [−3.21, 5.16]	−1.963 [−6.49, 2.56]	−0.632 [−5.63, 4.36]	−4.498 [−14.56, 5.57]	−0.606 [−11.38, 10.17]

*Note*. SOP = sense of place; PS = place attachment; PI = place identity, PD = place dependence; CI = confidence interval; UB = unadjusted model; AB = adjusted model; Ref = reference group.

Adjusted for control variables with a p < .10.

a Statistically significant outcomes at * *< .05, *p *<  .01, or *p *<  .001.

## 3 RESULTS

### 3.1 Descriptive statistics

As shown in Table 1, participants showed a moderate mean score of SOP on a scale of 1 to 5. Overall, participants showed stronger levels of general SOP (mean [*M*] = 3.082, standard deviation [*SD*] = 0.517). In terms of the individual SOP domains, participants seem to have stronger PA (*M* = 3.224, *SD* = 0.803) and PI (*M* = 3.167, *SD* = 0.723) than PD, which was fairly average, with a mean score of *M* = 2.963 and *SD* = 0.749. Further descriptive data are summarized in Table 1.

### 3.2 SOP and migration factors

As shown in Table 2, even after controlling for length of stay, language proficiency, age, gender, place of origin, PD was positively associated with region of residence (b = 1.26, 95% confidence interval [CI] [0.26, 2.25], p = 0.014) and was higher among African residents living in the western region of the Netherlands compared with those living in the northern region.

In addition, PD was lower among participants having a little knowledge of the Dutch language (b = −0.99, 95% CI [−1.99, −.01], p = 0.051) compared to those speaking it fluently. The main effects of length of stay were positively associated with several place experiences such as PD (b = 1.65, 95% CI [.77, 2.52], p = 0.000), PA (b = 1.43, 95% CI [.47, 2.78], p = 0.004), and general SOP (b = 2.99, 95% CI [.87, 5.12], p = 0.006). Stronger SOP effects were reported among long‐term African residents (more than 5 years) compared with short‐term residents (less than 5 years). PI scores were not significantly related to any migration factor, nor was immigration status found to be associated with any SOP experience.

### 3.3 Place and sociodemographic factors

Univariate and multivariable analyses of SOP experiences and sociodemographic factors are summarized in Table 2. First, PD was more positively related to age such that PD in African residents aged 46 years and older (b = 1.57, 95% CI [0.06, 3.08], p = 0.042; b = 1.759, 95% CI [.17, 3.34], p = 0.030) was stronger than PD in residents younger than 45 years of age. PD was also significantly lower among females than males (b = −1.18, 95% CI [−2.07, −0.30], p = 0.009). This negative relationship was retained even after adjusting for covariates. In addition, scores of PA (b = −1.37, 95% CI [−2.32, −.42]. p = 0.005) and general SOP (b = −2.57, 95% CI [−4.70, −.45], p = 0.018) were significantly related to education background and were lower in African residents with a tertiary educational background (albeit these relationships did not remain significant after controlling for covariates).

Furthermore, PD was statistically related to region of origin and was higher among residents from Western Africa (b = 20.6, 95% CI [0.24, 3.89], p = 0.027) compared with those from Central Africa. This result remained statistically significant even in the adjusted model controlling for region of residence, length of stay, Dutch language proficiency, age, and gender. A similar significant relationship was found between region of origin and general SOP; in both models, score ratings were higher for residents from Western Africa than those from Central Africa (b = 4.819, 95% CI [.23, 9.41], p = 0.040).

In addition, there was no relationship between participants’ religious background and SOP experiences. However, there was a positive association between PD and religious background when controlled for region of residence, length of stay, Dutch language proficiency, age, and gender. This result may suggest the mediating role of migration and sociodemographic factors among religious participants who depend on Dutch society. There was no statistically significant correlation between relationship status and any SOP experience, or between PI and any sociodemographic variable, nor was a statistically significant relationship found between education and PD.

## 4 DISCUSSION

We examined the association between migration and sociodemographic factors and SOP in cross‐sectional data of 175 sub‐Saharan African residents living in the northern and western regions of the Netherlands. Here is a summary of our main findings:

- Region of residence was related to dependence on Dutch society: PD was stronger among African residents in the Western Netherlands than those in the Northern Netherlands.
- Region of origin was associated with dependence on Dutch society: Participants from Western Africa had stronger levels of PD compared to those from Central Africa.
- Language proficiency was associated with dependence on Dutch society: Participants with a little knowledge of Dutch had lower PD than those who can speak Dutch fluently.
- Age differences were related to attachment to and dependence on Dutch society: Compared with younger people, older adults are more likely to develop PA and have PD.
- Length of stay is related to attachment to and dependence on Dutch society: PA and PD are stronger among long‐term African residents (more than 5 years) compared with short‐term residents (less than 5 years).
- Educational background is associated with attachment to Dutch society: PA is weak among African residents with a tertiary educational background compared to those with a high school education or no educational qualification.
- PD was weaker among female participants compared with their male counterparts.

In H1, we proposed that SOP experiences will be associated with migration‐related factors. We were able to confirm this hypothesis in relation to the region of residence, length of stay, and language proficiency. First, length of stay was related to SOP in terms of PD and PA. These SOP experiences were stronger among long‐term residents (more than 5 years) than with short‐term residents (less than 5 years). These findings could be a result of the benefits of being a long‐term resident, such as access to the health care system, social contacts, and other opportunities. These resources give long‐term residents better and more meaningful SOP than those enjoyed by short‐term residents, who may struggle to form a sense of community and access opportunities in the Netherlands. This corroborates the findings of Heleniak (2009) who argues that the differences in SOP may come about as a result of a weak sense of community and issues related to place of residence.

This was further revealed in our study, showing that dependence (PD) on the Netherlands among African residents was related to *locality* and was stronger among those living in the Western Netherlands, which is more metropolitan and multicultural (with cities such as Amsterdam, Rotterdam, Leiden, Utrecht, Den Haag) than the Northern Netherlands, which is less metropolitan and multicultural (with cities such as Groningen, Emmen, Assen). This suggests that African residents living in the western part of the Netherlands depend on the activities, resources, and attributes of that region that may be offering them a sense of security, value, and ascribed meanings (Stokols & Shumaker 1981).

In addition, PD was inversely related to the language proficiency among African residents with a little knowledge of Dutch, suggesting a link between speaking the language of the place and the value ascribed to that place (Tuan, 1991). This negative relationship could suggest that the lesser proficiency with the Dutch language, the more likely it is for the participants to depend on the Dutch society. This could also mean that participants are likely to depend on various place resources or activities (e.g., Dutch language classes, cultural orientation, Dutch events) they rely on to learn about the Dutch society and develop their Dutch‐speaking skills because of their little knowledge of the Dutch language. The link between Dutch language proficiency and SOP still requires further research to gain clarity.

In H2, we also estimated that SOP experiences will be related to sociodemographic factors. We were able to confirm H2 for sociodemographic variables such as age, gender, region of origin, level of education, and religious background. The main effects of age differences among older African residents (aged 46 years and older and most of whom have been residents for more than five years) were related to PD and PA. This result suggests that older adults (aged 46 years and older) are likely to have a stronger SOP than their younger counterparts (aged 18–45 years). This corroborates the findings of the study by Wiles, Leibing, Guberman, Reeve, and Allen (2012) on “aging in place,” in which age was seen as an advantage in terms of developing a “sense of attachment or connection and feelings of security and familiarity in relation to both homes and communities” (p. 357). According to Wiles et al. (2012), the more advanced one is in a place, the more likelihood there is that the person will strengthen their SOP through their network of relationships and roles in the place.

Gender was also related to PD, with female African residents being less dependent on the Netherlands than their male counterparts, thus corroborating the work of McCarthy (2013), who reasons that place can be a setting “within which gender relations may be reinforced, renegotiated or deeply embedded” (p. 77). The role of gender differences in SOP also suggests that affiliative needs and exploration curiosity are related in complex ways to the socialization of gender roles and mobility (Frieze & Li, 2010).

Our study also reveals the negative relationship between level of education and PA, with weak attachment to Dutch society expressed by educated African residents compared to those with a high school education or less educational attainment. This result may fit into several interpretations. One interpretation is that the more educated the participants become in Dutch society, the more likely it is for them to experience place dissatisfaction or fewer ties to Dutch society because of a lack of opportunities in the areas of their qualification, compared with those who are less educated and willing to take any job offer,.

Another interpretation could be the effect of transnationalism, which may be fostered by a higher education, where the labor market is global rather than local, in that the education obtained in developing African countries is often not recognized by employers in the Netherlands. Other issues to consider are workplace discrimination including expecting to produce twice or more than Dutch counterparts to be recognized or promoted and occupying positions not commensurate with their qualification. Educated migrants may also tend to keep strong ties with their home country to maximize their chance of returning to high positions and better jobs or increased political involvement. A study by Nekby, Rodin, and Ozcan (2009) suggests that this sense of place dissatisfaction is stronger in men than women.

We further argue that this sense of place dissatisfaction or withdrawal from Dutch society among educated migrants is likely to be because of the education–occupation mismatch (Villarreal, 2017). Compared with low‐educated migrants, it is expected that educated migrants benefit less from migration because of their economic disadvantage and lack of opportunities in the fields of their educational qualification, often a result of racial and sociodemographic backgrounds (e.g., Djamba & Kimuna, 2011) and employment hierarchies associated with ethnicity (Friberg & Midtboen, 2017), among other reasons. This particular finding corroborates several other studies examining the link between PA and education (e.g., Lewicka, 2010; Maliepaard, Lubbers, & Gijsberts, 2010) and is well documented in the education–occupation mismatch literature (e.g., Hartog, 2000; Quintini, 2011; Villarreal, 2017). The link between SOP and educational attainment warrants further inquiry.

A limitation of the study is the cross‐sectional design, which may have made it impossible to make causal inferences, although some of the findings are evocative. Some further directions are worth mentioning: SOP among African migrants in other parts of the Netherlands, other factors related to SOP such as religious affiliation, and interactive influences between sociodemographic status and migration factors on SOP variables.

### 4.1 Conclusion

In conclusion, migration and sociodemographic factors remain important variables for discussing the SOP narrative of African migrants, as shown in the study results. Sense of place appears to be a very important, understudied theme in the diasporic experience of Africans in the Netherlands, one related to their place of residence, region of origin, Dutch language proficiency, length of stay, age differences, gender, and educational background. It would appear that level of education is an important future research in terms of understanding the dynamics of SOP among the African diasporas, even though we argued that the weak SOP among educated migrants is likely to be as a result of their education–occupation mismatch and economic disadvantage in an international labor market where race and ethnicity matter. It is also important not to brush over the differences between northern and western regions of the Netherlands because the regions indicate something about cultural contact and the role of social diversity in promoting SOP among non‐natives. Adequate attention should be given to the effects of these sociodemographic and migration variables on SOP experiences because this could help to foster a sense of social belonging, identity, and naturalization in African migrants in Europe.
